# Analysis of temporal expression profiles after sciatic nerve injury by bioinformatic method

**DOI:** 10.1038/s41598-017-10127-1

**Published:** 2017-08-29

**Authors:** Yichong Zhang, Yuanbo Zhan, Na Han, Yuhui Kou, Xiaofeng Yin, Peixun Zhang

**Affiliations:** 1 Peking University People’s Hospital, Beijing, China; 2 The Second Affiliated Hospital of Harbin Medical University, Harbin, China

## Abstract

After Peripheral nerve injuries (PNI), many complicated pathophysiologic processes will happen. A global view of functional changes following PNI is essential for the looking for the adequate therapeutic approaches. In this study, we performed an in-depth analysis on the temporal expression profiles after sciatic nerve injury by bioinformatic methods, including (1) cluster analysis of the samples; (2) identification of gene co-expression modules(CEMs) correlated with the time points; (3) analysis of differentially expressed genes at each time point (DEGs-ET); (4) analysis of differentially expressed genes varying over time (DEGs-OT); (5) creating Pairwise Correlation Plot for the samples; (6) Time Series Regression Analysis; (7) Determining the pathway, GO (gene ontology) and drug by enrichment analysis. We found that at a 3 h “window period” some specific gene expression may exist after PNI, and responses to lipopolysaccharide (LPS) and TNF signaling pathway may play important roles, suggesting that the inflammatory microenvironment exists after PNI. We also found that troglitazone was closely associated with the change of gene expression after PNI. Therefore, the further evaluation of the precise mechanism of troglitazone on PNI is needed and it may contribute to the development of new drugs for patients with PNI.

## Introduction

Peripheral nerve injury (PNI), one of serious health problems, can often lead to lifelong disability^1^. Despite many preclinical and clinical studies have made significant progress in understanding the mechanism underlying this disease^2^, many complicated pathophysiology processes will happen, including a series of cellular and molecular responses accompanied with the alteration of various gene expressions after PNI^3^. The poorly understanding brings certain difficulties in searching for the adequate therapeutic approaches^4^. Thus, a global perspective of changes following PNI is warranted. Microarray is one of the most popular methods that can detect the genome-wide transcriptome profiling in certain conditions. Some studies^5–9^ have identified many genes that are disturbed after peripheral nerve injury. However, the precise mechanisms underlying the specific events or biological processes after PNI are not completely understood. For the occurrence and development of PNI, it is well known that genes are not alone and usually act through joint actions with other genes in pathways or networks. Thus, one of the interesting questions can be aroused, what’s the particular function and pathways changed in the process of PNI? And which drug can be potentially used for the treatment of PNI? To preliminarily answer those questions, the high-throughput gene data related to PNI can be explored conveniently and time-saving. In the other hand, as Li *et al*.^10^ mentioned, despite high-throughput gene data are expanding and can be obtained quickly, the in-depth and comprehensive analysis remains to be completed by the aid of newly-developed statistical and bioinformatic tools. Moreover, by these bioinformatic methods, the different perspective and valuable information about the molecular regulation of transcriptional responses of PNI will be disclosed.

Sciatic nerve injury is a widely used model for PNI and peripheral nerve regeneration studies^10^. The anatomy of the dynamic changes of differentially expressed genes associated with PNI can help understand the response in the process of the PNI, and find some new treatment strategies target the regulation of essential genes. To date, temporal expression profiles or time course data for sciatic nerve injury have been published^11^. Therefore, the aim of this study was to in-depth analyze the temporal expression profiles after sciatic nerve injury by bioinformatic methods. The second aim of this study was to elucidate the biological process and pathways in the response of sciatic nerve injury.

The analysis process is composed of the following seven parts: (1) cluster analysis of the samples in the microarray data; (2) identification of gene co-expression modules (CEMs) correlated with the time points; (3) analysis of differentially expressed genes at each time point (DEGs-ET); (4) analysis of differentially expressed genes varying over time (DEGs-OT); (5) creating Pairwise Correlation Plot for the samples in the microarray data; (6) Time Series Regression Analysis; (7) determining the Pathway, GO and drug by enrichment analysis.

## Materials and Methods

### Microarray Data

The gene chip data of GSE33175^11^ was obtained from National Center of Biotechnology Information Gene Expression Omnibus (GEO) database^12^. The more detail information about GSE33175 can refer to Wang *et al*.^11^. Briefly, the platform used in GSE33175 was GPL7294 Agilent-014879 Whole Rat Genome Microarray 4 × 44K G4131F. The experiments were conducted on adult male Sprague-Dawley rats weighing 180–220 g. Proximal sciatic nerve tissues (0.5 cm) were generated at 0 h, 0.5 h, 1 h, 3 h, 6 h and 9 h after sciatic nerve resection. Total RNA extracted from those tissues was used for cDNA array hybridization^11^.

### Statistical analysis of microarray data

#### Data pre-processing

Gene chip data of GSE33175 were analyzed by using BRB Array Tools (version 4.5.1 and R version 3.2.5; http://linus.nci.nih.gov/BRB-ArrayTools.html)^13^. The raw expression data were converted to log2 values and then normalized by using the quantiles normalization. In terms of spot filters, spots with intensity <10 were removed. The replicate spots within an array were averaged. Moreover, the genes under any of the following conditions were excluded: percentile of the log-ratio variation in less than 75, percent of data missing or filtered out exceeds 50%.

### Cluster analysis of the samples

Clustering samples were performed using the entire set of genes that pass the aforementioned filter levels. Hierarchical clustering was carried out using centered correlation and average linkage^13, 14^.

### Identification of gene co-expression modules (CEMs) correlated with the time point

To identify CEMs, the weighted correlation network analysis (WGCNA)^15^ was used. The remarkable characteristics of WGCNA is to finding clusters (modules) of highly correlated genes, for summarizing such clusters using the module eigengene for relating modules to external sample traits. The network and the modules were constructed and detected by using WGCNA as previously described^15, 16^. The name of co-expression modules were named as the colour assigned by WGCNA. The more detail information including script and parameters can be found in Supporting Information S1.

### Analysis of differentially expressed genes

#### Analysis of differentially expressed genes at each time point (DEGs-ET)

GSE33175 data include six experimental groups at different times. To identify the DEGs-ET at 0.5 h, 1 h, 3 h, 6 h and 9 h comparing with the time point 0 h, a random-variance t-test was used. Genes were considered highly significant if their P value was less than 0.001 and the false discovery rate (FDR) less than 0.05.

#### Analysis of differentially expressed genes varying over time (DEGs- OT)

To identify the genes whose expressions were varying over time (DEGs-OT), the plug-in “Time course analysis” from BRB Array Tools was employed. This plugin can be used for regression analysis of time series expression data. The tests were performed at a FDR threshold of 0.01^13^.

### Determining the relationship of the expression of arrays between different time points

To determine the relationship of the expression of arrays between different time points, we performed a correlation analysis for the expression of arrays using the Spearman Correlation Test by using the plug pairwise correlation plot in BRB Array Tools. Log intensity of the top 50 genes from the DEGs-OT was utilized in this process.

### Time series regression analysis

To identify the genes or gene sets co-regulated time dependently (temporal expression profiles)^17^, software program “Short Time-series Expression Miner” (STEM, version 1.3.9, http://www.cs.cmu.edu/~jernst/stem/) was used^18^. The main advantage of STEM is that it implements a novel method for clustering short time series expression data that can differentiate between real and random patterns^18^. To reduce the redundancy, the genes whose expressions were varying over time were used in this process. The raw expression data of these genes from the different times (0 h, 0.5 h, 1 h, 3 h, 6 h, and 9 h) were retrieved by BRB Array Tools, and then sorted by the user manual of STEM. Finally, STEM Clustering Method was adopted to cluster genes with the parameters by default^18^. The group was defined as temporal expression profiles n (n = 1,2,3…n) according to the corresponding P values. Genes belong to the profile n were fellow named temporal expression profiles gene n (TEPGs).

### To determine the pathway, gene ontology (GO) and drug by enrichment analysis

We thought the common gene disturbance measured by different methods could help us understand the molecular mechanism of PNI. Therefore, it is reasonable to presume that the same GO term, pathways and drugs identified in four datasets (CEMs, DEGs-ET, DEGs-OT, and TEPGs,) might be closely related to PNI.

Since we obtained four different gene sets (CEMs, DEGs-ET, DEGs- OT and TEPGs), we performed a compared enrichment analysis. Simply, the identified CEM, DEGs-ET, DEGs-OT and TEPGs were then used to obtain functional pathways and GO term. Relevant GO terms for those gene set were analyzed by using Funrich software (http://www.funrich.org)^19^. Enriched pathways and drugs were identified by using ToppCluster (https://toppcluster.cchmc.org/)^20^. The functional annotations for the GO term level were mainly focused on biological process in this study. Hypergeometric test and multiple-testing corrections (Bonferroni) were employed in this process with p ≤ 0.05 considered significant.

## Result

### Clustering samples

**Figure 1:**
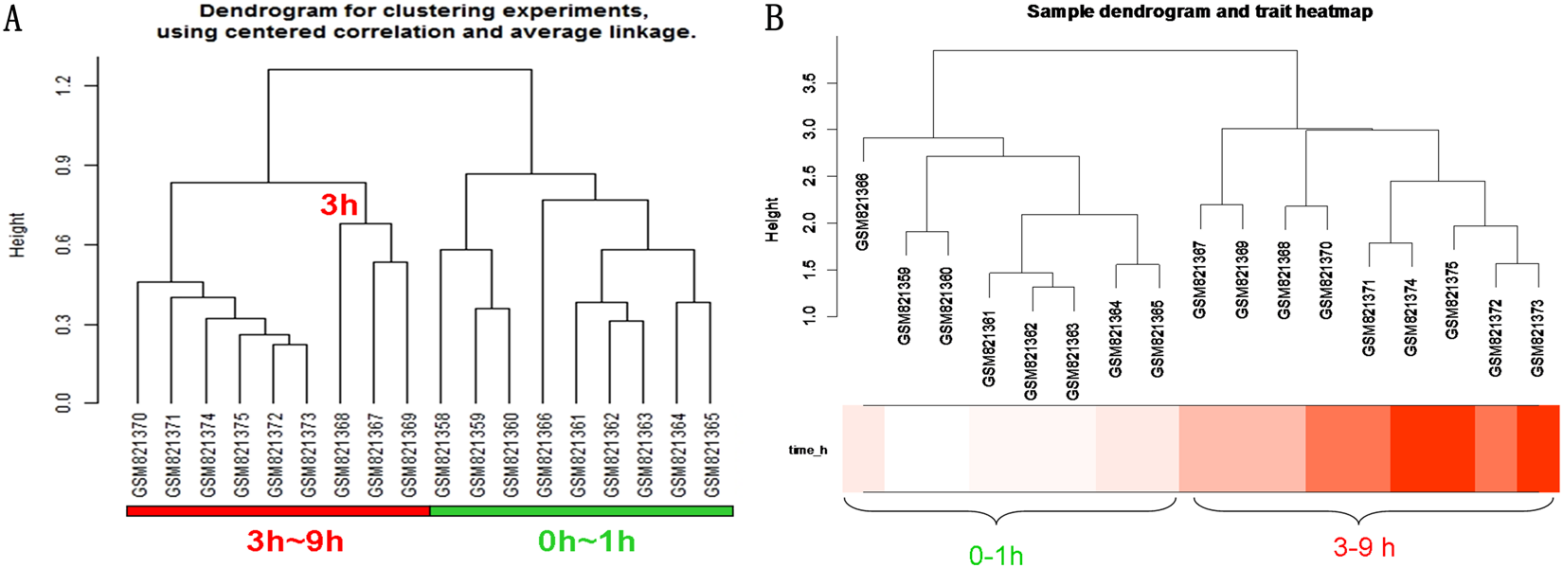
Clustering samples for GSE 33175 (**A**), Clustering by WGCNA; (**B**), Clustering by BRB Arraytools. 0 h: GSM821358, GSM821359, GSM821360; 0.5 h: GSM821361, GSM821362, GSM821363; 1 h: GSM821364, GSM821365, GSM821366; 3 h: GSM821367, GSM821368, GSM821369; 6 h: GSM821370 GSM821371, GSM821372; 9 h: GSM821373, GSM821374, GSM821375.

We used hierarchical clustering to cluster the samples. The results showed that samples of proximal sciatic nerve stratified into two main groups (Fig. 1A). Samples at 0 h, 0.5 h, and 1 h were distinctly separated from those at 3 h, 6 h and 9 h (Fig. 1A).

### To partition the gene set into co-expression modules (CEMs)

**Figure 2:**
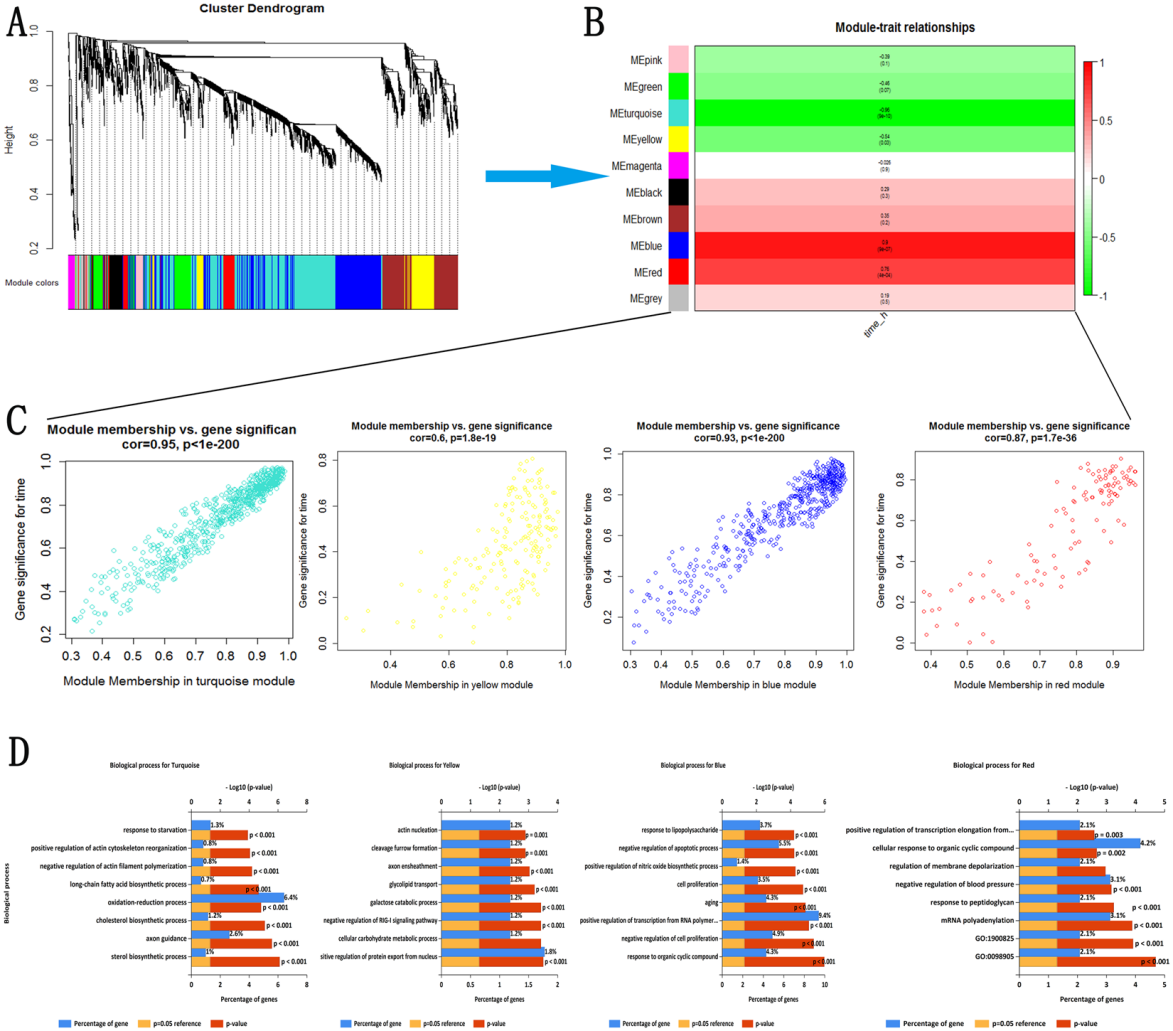
Four modules that significantly associated with the time point. (**A**) Gene dendrogram showing the co-expression modules constructed by the WGCNA and labeled by colors; (**B**) Module-trait associations. Each row contains the corresponding correlation and p-value. And each of them was color-coded by correlation according to the color legend; (**C**) A scatter plot of gene significance (GS) for time vs. module membership (MM) in the turquoise, yellow, blue and red module. There were highly significant correlations between GS and MM in those modules; (**D**) Enriched biological processes for those modules.

By using the WGCNA, we performed a sample clustering to see if there are any obvious outliers. We found that the outline of the samples clustering was similarity with that drawn from the BRB Array Tools (Fig. 1B). In addition, four modules that significantly associated with the time point were identified (Fig. 2 and Table S1).

### Differentially expressed genes

#### Differentially expressed genes at each time point (DEGs-ET)

**Figure 3:**
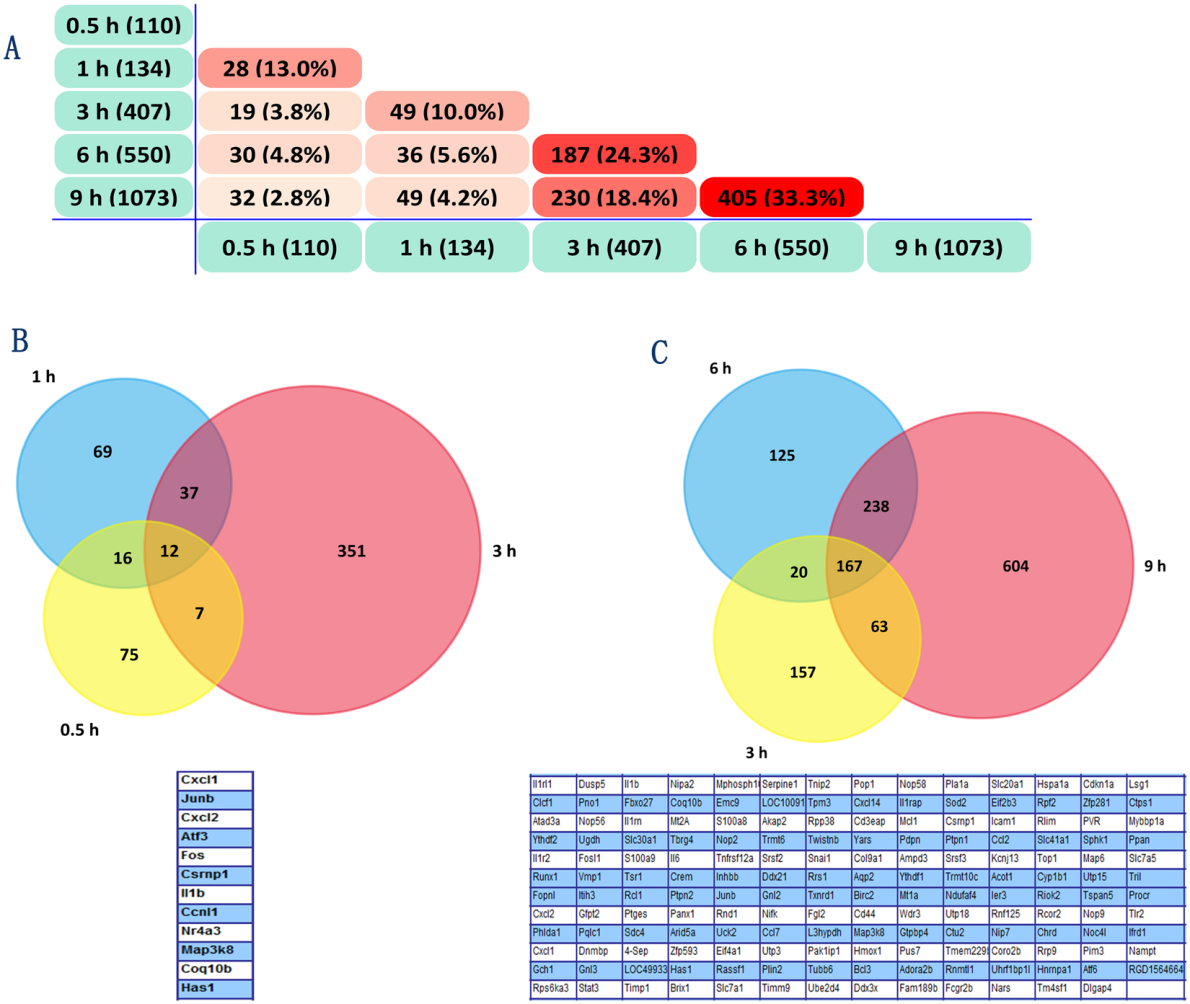
Overlapped genes between different classes’ comparisons (Time points). (**A**) the overlapped DEGs between different point had an apparent boundary after 3 h; (**B**,**C**) the number of overlapped DEGs between 0.5 h, 1 h and 3 h(12 genes) were less than the overlapped gene numbers between 3 h, 6 h and 9 h(167 genes).

After data pre-processing and class comparison between different time points, 110 genes (0.5 h vs 0 h), 134 genes (1 h vs 0 h), 407 genes (3 h vs 0 h), 550 genes (6 h vs 0 h), and 1073 genes (9 h vs 0 h) were identified, respectively (Tables S2–S6). The overlapped genes between different classes’ comparisons were shown (Fig. 3).

#### Differentially expressed genes varying over time (DEGs- OT)

**Figure 4:**
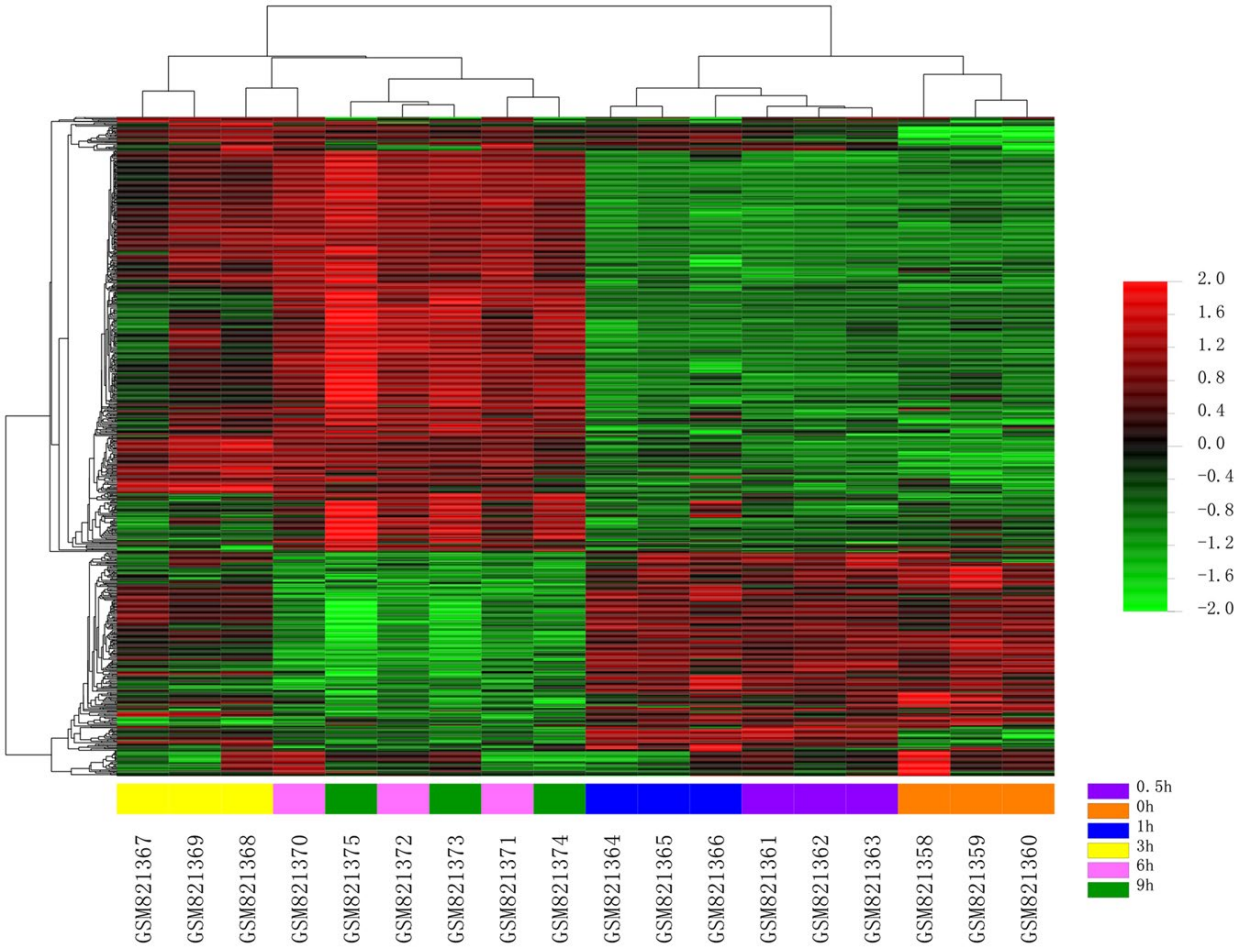
Heat map of 570 genes expressed varying over time (0 h to 9 h). By using the plug-in “Time course analysis” from BRB Array Tools, the DEGs crossed over time have also displayed a remarkable diversion at 3 h.

**Figure 5:**
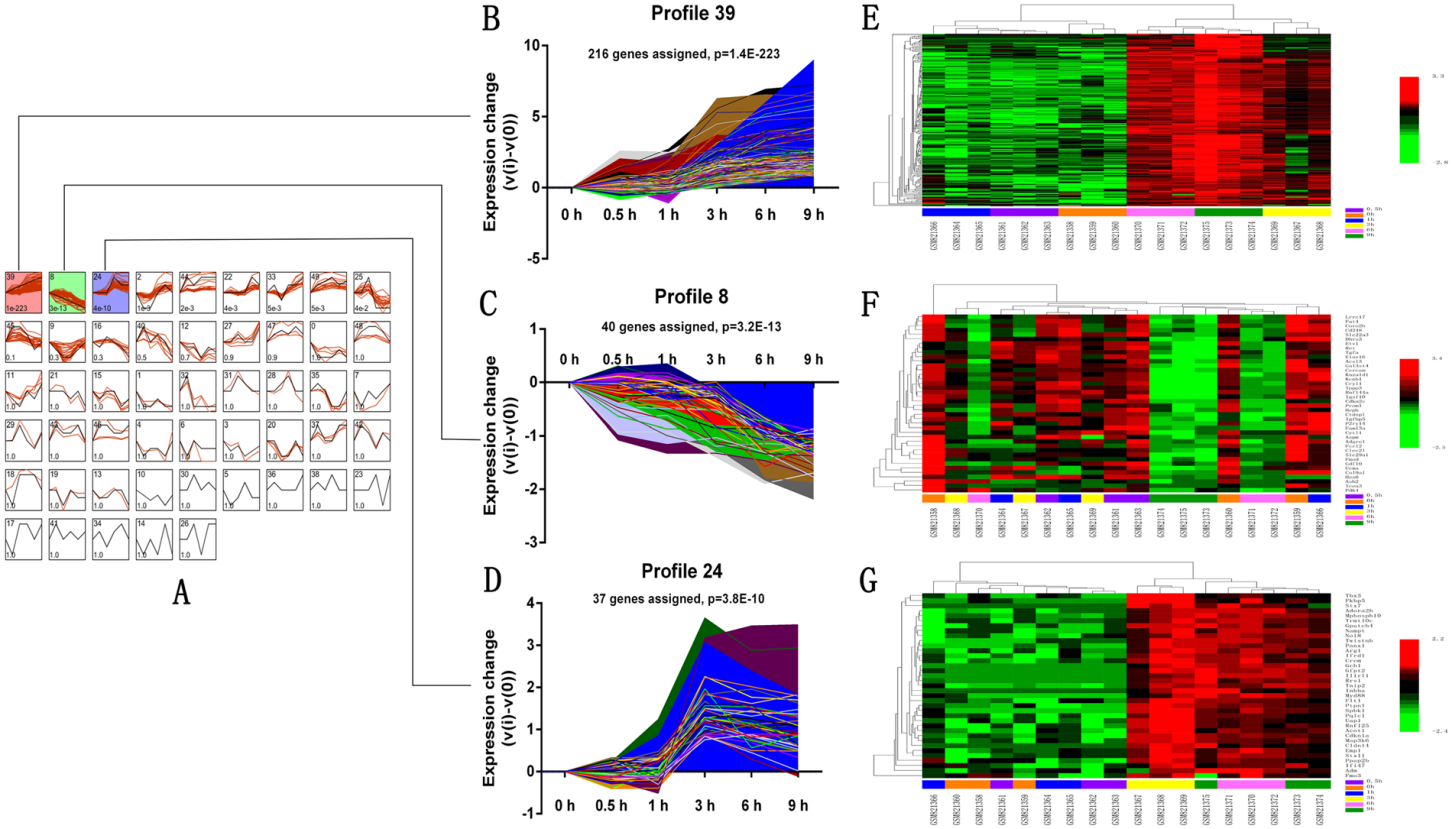
Three temporal expression profiles. The profile 39 and 24 displayed increased mRNA expression after sciatic nerve resection. However, profile 8 demonstrated a decreased mRNA expression patterns after sciatic nerve resection. The three expression patterns have a similarly remarkable turning point at 3 hours. (**A**) Among the 49 model temporal expression profiles, three temporal expression profiles (profile 39, 8 and 24) were statistically significant. (**B**,**C** and **D**) Enlargement of profile 39, 8 and 24 respectively. (**E**,**F** and **G**) Heat map of the profile 39, 8 and 24, respectively.

Firstly, after analysis the data GSE33175 by the plug-in “Time course analysis” from BRB Array Tools, 570 genes expressed varying over time (0 h to 9 h; Table S7, Fig. 4). Moreover, after submitting the 570 DEGs- OT into the “Short Time-series Expression Miner” (STEM, version 1.3.9, http://www.cs.cmu.edu/~jernst/stem/), they were totally mapped to 49 model temporal expression profiles, three temporal expression profiles (profile 39, 8 and 24) were statistically significant (Tables S8–S10). The profile 39 and 24 displayed increased mRNA expression after sciatic nerve resection. However, profile 8 demonstrated a decreased mRNA expression pattern after sciatic nerve resection. More interestingly, all the expression patterns have a remarkable turning point at 3 h (Fig. 5).

### Relationship of gene expression arrays between different time points

**Figure 6:**
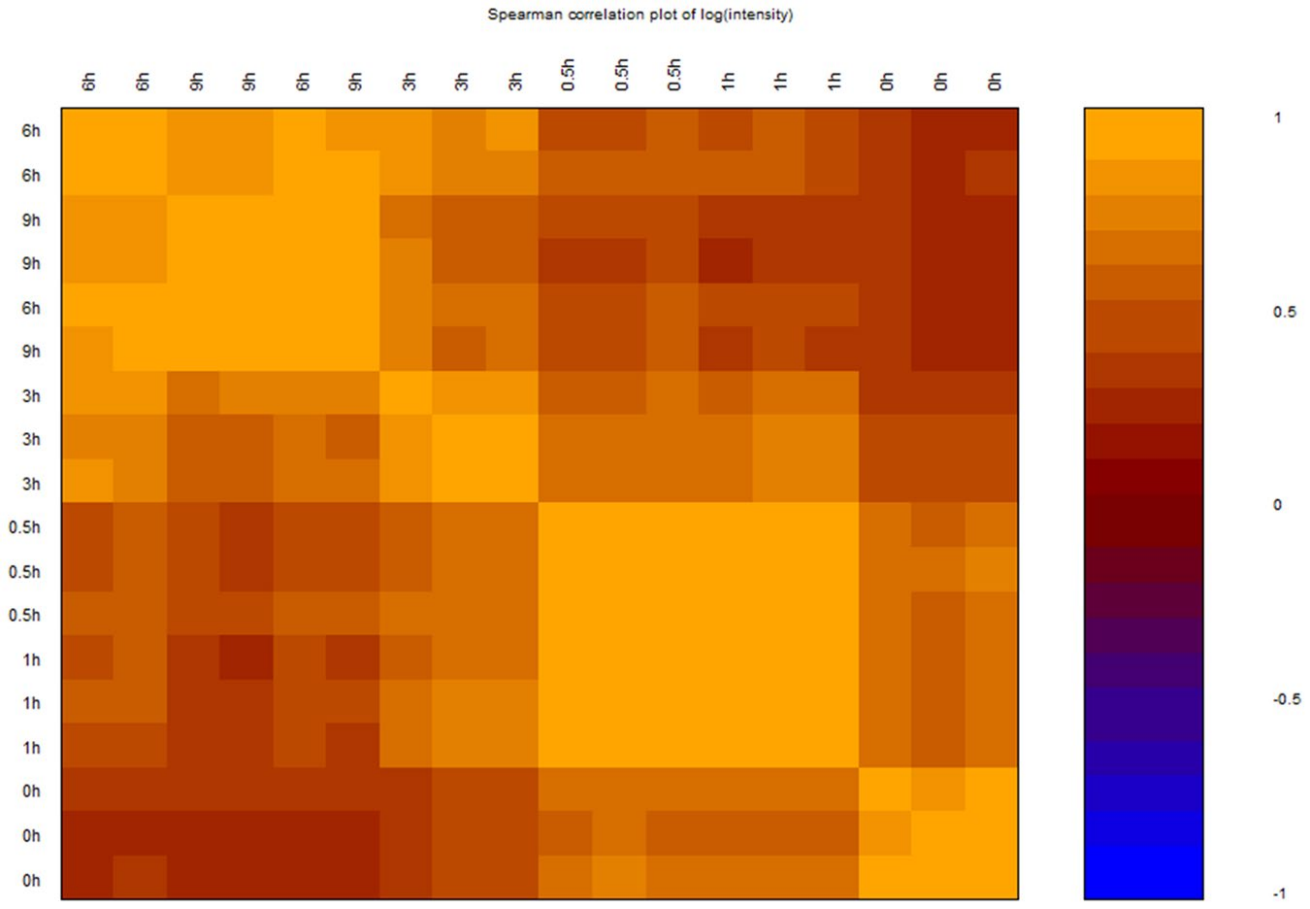
Pairwise Correlation Plot for different time point. Image plot of correlation of expression for the arrays (arrays are ordered based on the hierarchical clustering using the average linkage method).

By using the plug pairwise correlation plot in BRB Array Tools, Pairwise Correlation Plot was created. The plot showed strong correlation between 0.5 h and 1 h. However, the correlation between 0.5 h or 1 h and 3 h or 6 h or 9 h was decreased (Fig. 6 and Table S11).

### Enrichment analysis

Furthermore, based on the four gene sets, CEMs, DEGs-ET, DEGs-OT, and TEPGs, we performed a functional enrichment analysis. Using the Funrich and ToppCluster, the enrichment GO term (biological processes), pathway and drugs were picked out.

### Pathways and GO enrichment analysis of the CEMs

As mentioned, four modules (labeled blue, red, turquoise, and yellow) that are highly associated with time had been identified. To facilitate a biological interpretation, we would like to know the function of the genes in the modules, whether they are significantly enriched in certain functional categories, pathways or which drugs can be potentially targeted.

After pathway enrichment analysis, we found that the turquoise module was mainly involved in metabolic pathways, such as cholesterol biosynthetic, super-pathway of cholesterol biosynthesis, cholesterol biosynthesis, steroid biosynthesis and mitochondrial fatty acid beta-oxidation. Only one pathway, galactose metabolism was enriched for the yellow modules. The blue module was enriched for genes related to rRNA modification in the nucleus and cytosol, Ribosome biogenesis in eukaryotes, rRNA processing in the nucleus and cytosol (Table S12). That may reflect that cholesterol metabolic might play an important role after the PNI.

Meanwhile, although few or no known biological processes were significantly enriched by those modules after the multiple-testing corrections (Bonferroni), the unadjusted results reminded that some biological processes, such as cell proliferation, response to lipopolysaccharide, positive regulation of nitric oxide biosynthetic process, negative regulation of apoptotic process, positive regulation of actin cytoskeleton reorganization, peripheral nervous system myelin maintenance might be associated with these modules (Table S13).

### Pathways and GO enrichment analysis of the DEGs-ET

**Figure 7:**
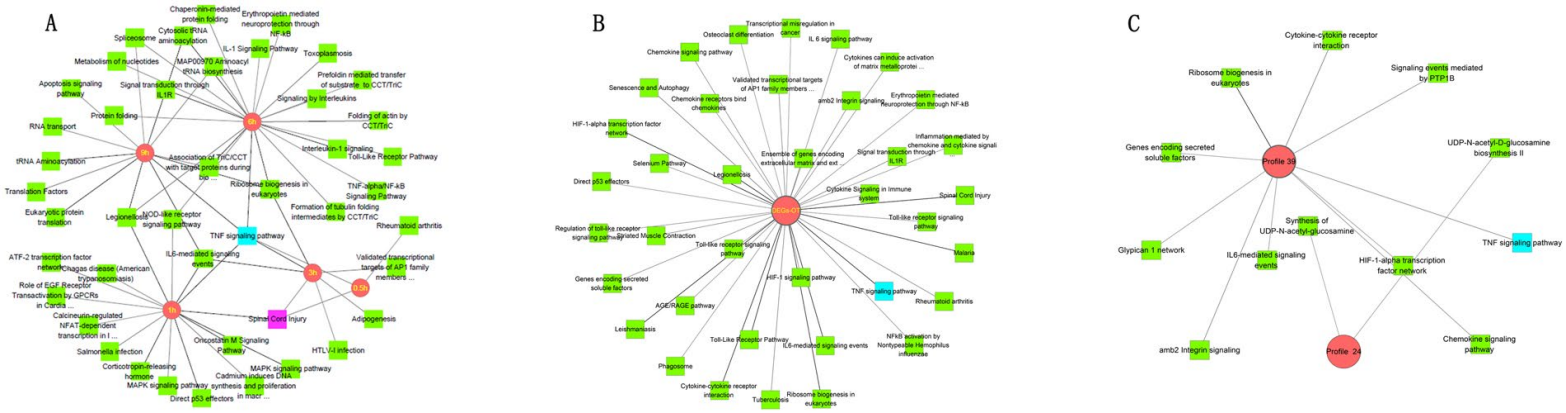
Enriched pathways associated with the DEGs-ET, DEGs-OT and TEPGs. The TNF signaling pathway (blue quadrate hub) was shown to be significantly enriched by all DEG﻿s from 0.5h, 1h, 3h, 6h and 9h. (**A**) Enriched pathways for DEGs-ET; (**B**) Enriched pathways for DEGs-OT; (**C**) Enriched pathways for the TEPGs. Quadrate hubs represent pathways, rounded hubs represent times.

We performed a comparative pathway enrichment analysis using ToppCluster, and the enriched pathways associated with the DEGs-ET were identified. As shown in Fig. 7A, three, sixteen, seven, twenty two and thirteen pathways were found to be enriched by DEGs-0.5 h, DEGs-1h, DEGs-3h, DEGs-6h and DEGs-9h, respectively (Table S14). Remarkably, only one pathway, the TNF signaling pathway was jointly enriched by DEGs-0.5 h, DEGs-1h, DEGs-3h, DEGs-6h and DEGs-9h (Fig. 7A). Also, the pathway of spinal cord injury was mutually enriched by DEGs-0.5 h, DEGs-1h and DEGs-3h. It suggests that some interactions may exist between the periphery and the central nervous system during the early phase after PNI.

**Figure 8:**
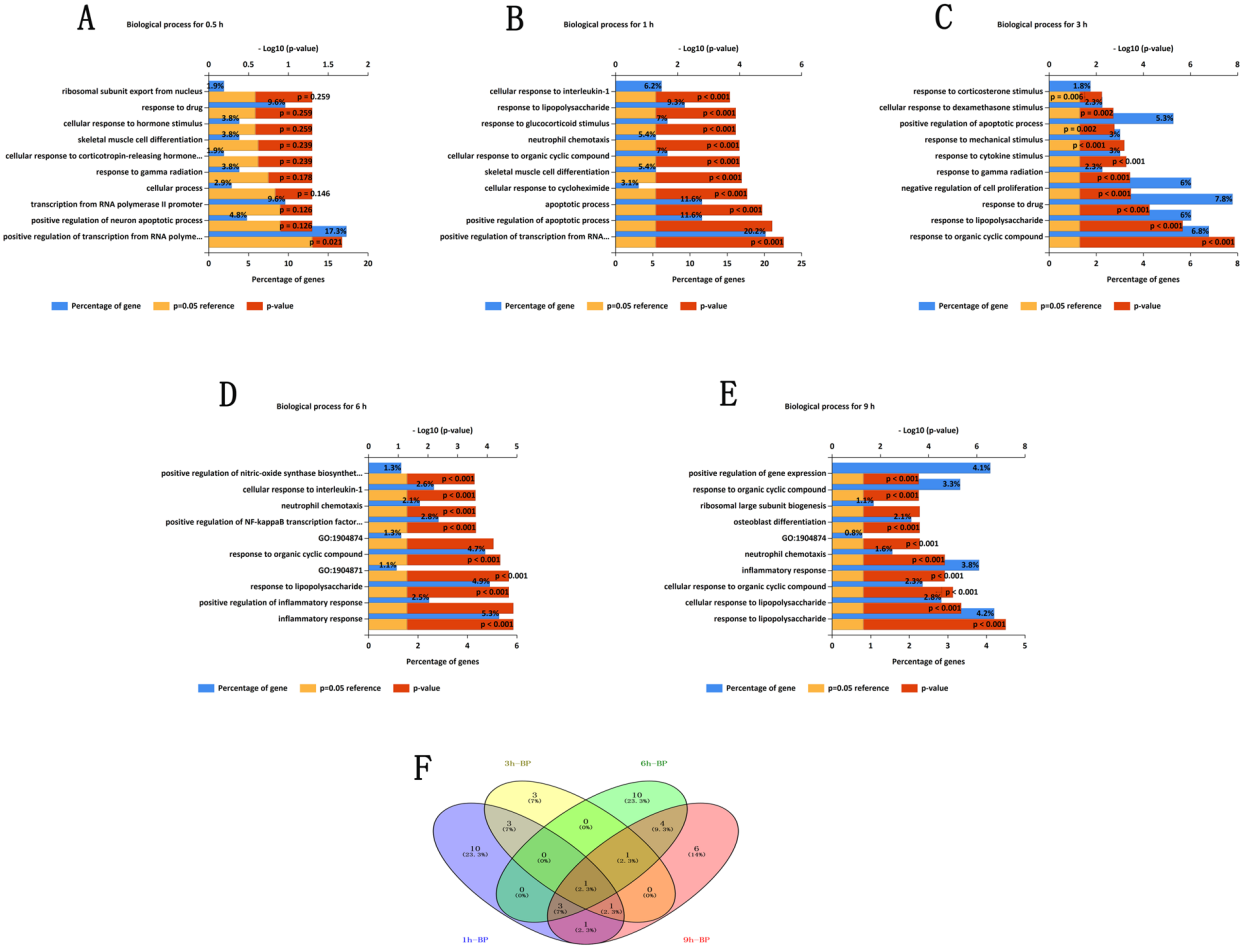
Biological processes for DEGs-ET (1h, 3h, 6h and 9h). ﻿(**A**,**B**,**C**,**D** and **E**)﻿ the enriched biological processes for 1h, 3h, 6h and 9h, respectively. (**F**) ﻿Venn diagram of the overlapped biological processes for 1h, 3h, 6h and 9h. One biological processes, lipopolysaccharide, had collectively enriched by all DEGs from 1h, 3h, 6h, and 9h.

According to the biological process analysis, there was only one significant biological process for DEGs-0.5 h: positive regulation of transcription from RNA polymerase II promoter (Table S15). DEGs-1h was mainly involved in apoptotic process, positive regulation of transcription from RNA polymerase II promoter, positive regulation of apoptotic process, inflammatory response, apoptotic process, cellular response to cycloheximide, skeletal muscle cell differentiation, inflammatory response, positive regulation of interleukin-8 production, response to mechanical stimulus (Table S16). DEGs-3h was mainly involved in response to organic cyclic compound, response to lipopolysaccharide, response to drug, negative regulation of cell proliferation, response to gamma radiation, response to cytokine stimulus, response to mechanical stimulus, positive regulation of apoptotic process, cellular response to dexamethasone stimulus (Table S17). DEGs-6h was mainly involved in inflammatory response, positive regulation of inflammatory response, response to lipopolysaccharide, response to organic cyclic compound, positive regulation of NF-κB transcription factor activity, neutrophil chemotaxis, cellular response to interleukin-1, positive regulation of nitric-oxide synthase biosynthetic process (Table S18). DEGs-9h was mainly involved in response to lipopolysaccharide, cellular response to lipopolysaccharide, cellular response to organic cyclic compound, inflammatory response, neutrophil chemotaxis, osteoblast differentiation, ribosomal large subunit biogenesis, response to organic cyclic compound (Table S19). In particular, the GO term, response to lipopolysaccharide was jointly enriched for DEGs-1h, DEGs-3h, DEGs-6h, and DEGs-9h (Fig. 8).

### Pathway and GO enrichment analysis of the DEGs-OT

**Figure 9:**
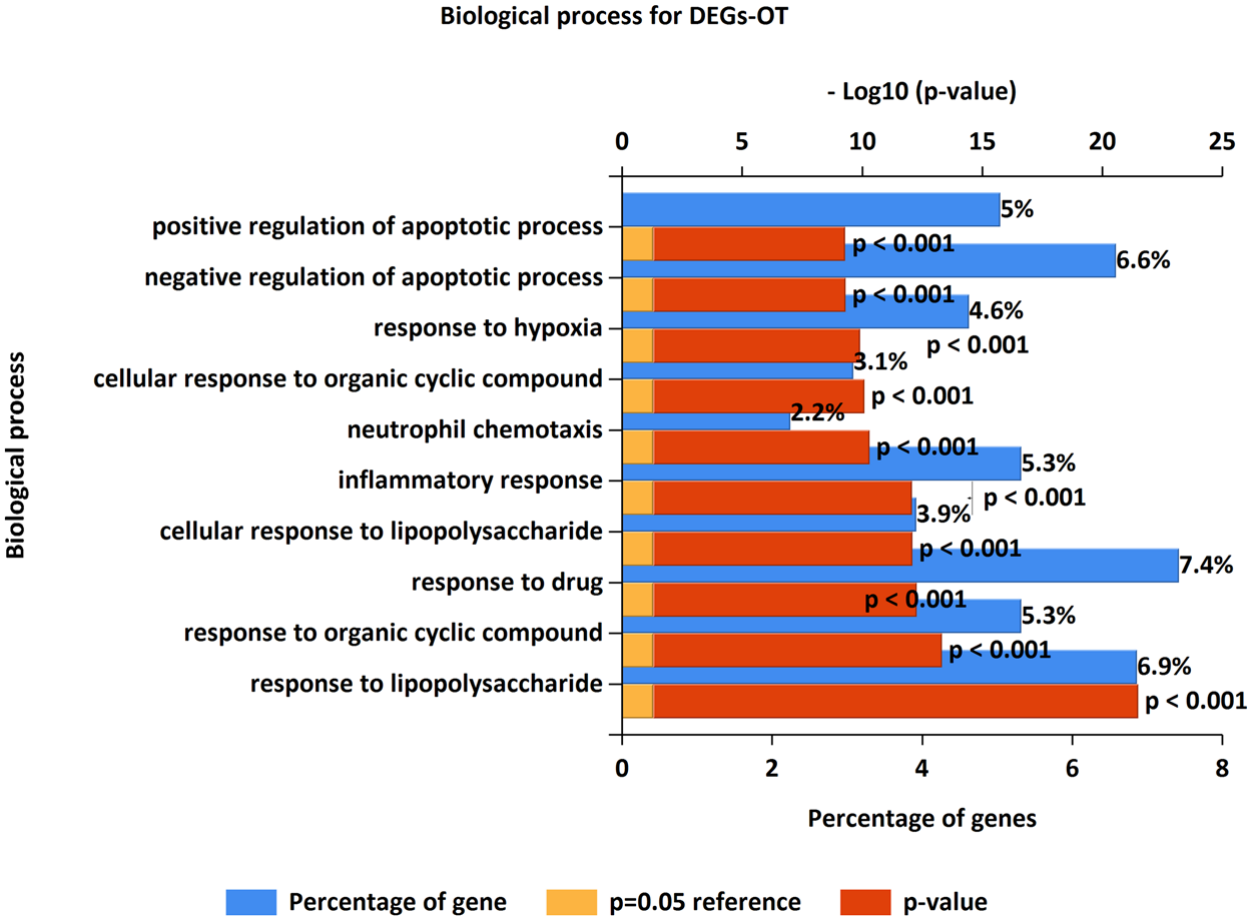
Top 10 Biological processes enriched by DEGs-OT.

For the DEGs-OT, 37 pathways were significantly enriched (Fig. 7B and Table S20), the top 10 pathways were HIF-1α transcription factor network, Toll-Like Receptor (TLR) Pathway, Ribosome biogenesis in eukaryotes, IL6-mediated signaling events, Malaria, Cytokine-cytokine receptor interaction, TNF signaling pathway, Spinal Cord Injury, Leishmaniasis, Legionellosis. And GO enrichment analysis revealed that the DEGs-OT was enriched in 58 biological processes (Fig. 9 and Table S21). Among them, some were primary implicated in inflammatory and immune process, such as response to lipopolysaccharide, inflammatory response, cellular response to lipopolysaccharide, neutrophil chemotaxis and immune response. Additionally, other non-inflammatory processes were also found to be related to DEGs-OT, such as response to mechanical stimulus, aging, response to cold, positive regulation of nitric oxide biosynthetic process, positive regulation of smooth muscle cell proliferation, response to heat, response to wounding, angiogenesis, response to axon injury, response to ischemia, muscle contraction, axonogenesis, negative regulation of smooth muscle cell proliferation, axon extension involved in axon guidance and regulation of axonogenesis.

### Pathway and GO enrichment analysis of the TEPGs

**Figure 10:**
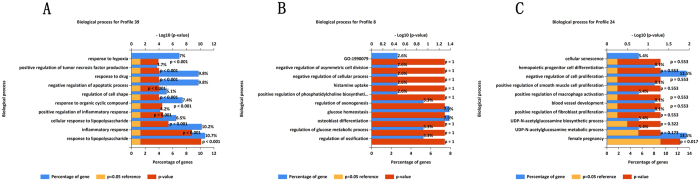
Biological processes enriched by TEPGs (profile 39, 8 and 24).

After enrichment analysis for TEPGs, 2 and 10 pathways were identified for the genes from profile 24 and 39, respectively (Fig. 7C and Table S22). However, the genes from profile 8 were not assigned with any pathway, and it may contain novel genes associated peripheral nerve injury. Enrichment analysis for biologically relevant processes showed the 19 and 1 biological processes were associated with profile 39 and profile 24, respectively. No biological process was founded for profile 8 (Fig. 10 and Tables S23–S25).

### Identification of drug

To determine which drugs may associate with the CEMs, DEGs-ET, DEGs-OT, and TEPGs, or which drugs can be targetable to interact with CEMs, DEGs-ET, DEGs-OT and TEPGs, the ToppCluster with its enrichment function was used. ToppCluster allows for analysis of enrichment against genes associated with various drug actions. After enrichment for the different gene sets, the results suggested that many drugs may have closed relationship with CEMs, DEGs-ET, DEGs-OT and TEPGs (Tables S26–S29). Comparing analysis of these results, we found that there was only one drug, troglitazone, were jointly enriched by CEMs, DEGs-ET, DEGs-OT and TEPGs, suggesting that troglitazone might be a potential drug for the treatment of PNI.

## Discussion

Peripheral nerve injuries (PNI) cause serious health problems, and there is no easily available formula for successful treatment^1, 21^. After PNI, a series of signal cascades can be triggered, accompanied with the alteration of various gene expressions^3^. In the past decade, the high-throughput technologies for gene analysis have tremendous potential in determining the pathogenesis of diseases, including the PNI. For example, by using the gene chip, a large number of differentially expressed genes are presented. However, the interpretation of what it means may be more significant for understanding the disease^22^. Moreover, bioinformatics has been developing rapidly and contributes to dissection of high-throughput data from novel perspectives.

Although the studies about the pathogenesis and etiology of PNI by using the high-throughput technology are beginning to emerge, the studies which explain the complex molecular mechanism based on the variety of levels are few. In the present study, we have used the various kinds of bioinformatics methods to detect the involved molecular functions and biological pathways after PNI. Moreover, the potential drug targets have also been explored.

### 3 h “window period” may exist for the gene expression after PNI

After nerve injury, injured nerve undergoes structural and molecular changes in preparation for the process of axonal regeneration^23^. A previous study suggested an “initial phase” for axonal regeneration^23^. In addition, clinical research showed that there was a significant negative correlation between the time interval from injury to surgery and motor function recovery, implying early exploration of sciatic nerve injuries can be beneficial if the nerve injury does not improve spontaneously^24^. However, the precise mechanism was still poorly understood. Moreover, the “initial phase” and “early” phase are always drawn from an empirical point of view and has no detailed timeline in short time points. Clinically, failure to diagnose early leads to permanent disability^25^. In the present study, the results suggest that the 3 h “time window” may give us new clues for further treatment of PNI.

Firstly, the results from the hierarchical clustering showed that samples from 0 h, 0.5 h, and 1 h were distinctly separate with the samples from 3 h, 6 h and 9 h (Fig. 1), reminding us that there is a turning point at 3 h. Secondly, we routinely analyzed the differentially expressed genes (DEGs) at each time point and the results showed that overlapped DEGs between different points had an apparent boundary after 3 h. As shown in Fig. 2, the overlapped gene numbers between 0.5 h, 1 h and 3 h were less than those between 3 h, 6 h and 9 h. Thirdly, by using the plug-in “Time course analysis” from BRB Array Tools, the DEGs crossing over time have also displayed a remarkable diversion at 3 h (Fig. 3). Fourthly, the plot showed strong correlation between 0.5 h and 1 h. However, the correlation between 0.5 h or 1 h and 3 h or 6 h or 9 h were decreased (Fig. 5). Lastly, by using the STEM, the expression of profiles 24, 8 and 39 have also shown a turning point at 3 h (Fig. 4).

### Responses to Lipopolysaccharide (LPS) and TNF signaling pathway play critical role after PNI

Previous studies have demonstrated that nervous system injury causes the almost immediate release of cytokines by glial cell and neurons^23, 24, 26^. Notoriously, spontaneous peripheral nerve regeneration is a frustratingly slow process. Many factors may have an impact on the effectiveness and success rate of the regeneration, including the degree of injury, time, distance, scar formation, end organ atrophy, distal nerve degeneration, and so on^27^. Understanding these progresses is a cornerstone for the development of new treatments in the future.

To further dissect mechanisms, unlike the previous analysis which performed GO and pathways analysis for differential genes found at six time-points, without comparing the GO and pathways between different time points, we used a comparative GO and pathway enrichment for the expression data related to PNI.

Interestingly, no matter which aspect of the gene set (except for profile 8 and 24) we used for enrichment analysis, it was indicated that “response to LPS” or “cellular response to LPS” always ranked among the top enriched GO terms (biological processes).

First of all, the unadjusted results from CEMs reminded that response to LPS might be associated with blue modules. Second, the biological process from GO analysis indicated that response to LPS has been collectively enriched by all DEGs from 1 h, 3 h, 6 h, and 9 h. What’s more, after exploring the associated GO terms in integrity across all over the time points (DEGs-OT) or partial profile (profile 39), “response to LPS” was also picked out.

LPS, a component of the outer membrane of Gram-negative bacteria, is a potent activator of innate immune responses. Due to the fact that LPS can induce endogenous inflammatory cytokines such as TNF-α, IL-1β and IL-6, which are responsible for the neurotoxicity observed in neurodegenerative diseases, LPS is always used as a proinflammatory agent to mimic neuroinflammation or neuropathic pain after PNI^28, 29^. Injury to the peripheral nervous system (PNS) can induce a well-orchestrated cellular process. Several inflammatory cytokines/chemokines are produced as early as 1 h after peripheral nerve lesion, with expression levels peaking at ~24 h^30^. Thus the enriched biological process in our study is consistent with the inflammatory microenvironment^30^, which indeed exists as early as 1 h after PNI (no significant inflammatory biological process was found at 0.5 h).

As to the pathway analysis, the TNF signaling pathway was shown to be significantly enriched by all DEGs from 0.5 h, 1 h, 3 h, 6 h and 9 h, suggesting an essential role of TNF signaling pathways in the early phase of PNI. This is consistent with results from a previous study^31^. As reported previously, TNF signaling pathway is implicated in the development of neuropathic pain after peripheral nerve injury^31^. TNF-α could differentially regulate synaptic plasticity in the hippocampus and spinal cord after PNI^32, 33^. Moreover, TNF mRNA had been detected early following PNI^31^.

### Troglitazone as a promising drug for the treatment of PNI

Besides, we have found troglitazone have a close relationship with CEMs, DEGs-ET, DEGs-OT and TEPGs. Troglitazone, one of the thiazolidinediones, was initially approved by FDA (Food and Drug Administration) of the United States but has subsequently been withdrawn from the market in the year 2000^34^. Later, other drugs with similar mechanism of action as troglitazone but presumably without liver toxicity were developed^34, 35^, such as rosiglitazone and pioglitazone.

To date, there was no record refer to the effect of troglitazone on PNI. However, the beneficial effect troglitazone on peripheral neuropathy in STZ-induced diabetic rats has been reported^36^. In addition, previous report showed that pioglitazone (also thiazolidinediones drugs) has a benefit on PNI. For instance, Reza *et al*.^37^ demonstrated that pioglitazone had a protective effect on sciatic nerve ischemia/reperfusion injury. Masaki *et al*.^38^ showed that pioglitazone could promote peripheral nerve remyelination.

At present, troglitazone cannot be clinically used in patients because of its deleterious effects on the liver. However, with the results from the present study (troglitazone was jointly enriched by CEMs, DEGs-ET, DEGs-OT and TEPGs), it is reasonable for us to presume that its potential bio-efficacy including on peripheral nerve injuries might be ignored. Thus it is possible that the evaluation of the precise mechanism of Troglitazone on PNI will lead to the development of new drugs for patients with PNI.

This study has limitations that should be acknowledged. Firstly, we only pay our attention on the short time after PIN, mainly within 9 h. More long time, such as couple of days should be considered in the future. Secondly, as similar to other study^39^, due to the analytical strategy in our study incorporated prior knowledge, some genes (in the modules or cluster) for which there are no functional interaction data available cannot be interpreted^39^. Finally, we combined various method and look into the overlapped pathway, GO and drugs for the PIN, that no doubt increased results’ reliability, and on other hand inevitably ignored some content, which needs great attention (we have put all of the results as additional material). Notwithstanding these limitations, we have provides a modular view for the PIN.

In conclusion, the time course gene expression was deeply analyzed by bioinformatics methods in this study. A 3 h “window period” for the gene expression after PNI provides a new clue for further treatment. Responses to LPS and TNF signaling pathway play an important role, suggesting an inflammatory microenvironment after PNI. Moreover, troglitazone is closely associated with the alteration of gene expression after PNI. Further evaluation of the precise mechanism of Troglitazone on PNI will lead to the development of new drugs for patients with PNI.

## Electronic supplementary material

Statistical code used for generating the weighted gene co-expression network.doc

Table S1

Table S2–S6

Table S7

Table S8–S10

Table S11

Table S12

Table S13

Table S14

Table S15–S19

Table S20

Table S21

Table S22

Table S23–S25

Table S26

Table S27

Table S28

Table S29
