# Supplementary material for: Analysis of temporal expression profiles after sciatic nerve injury by bioinformatic method
Source: Sci Rep. 2017 Aug 29;7:9818. doi: 10.1038/s41598-017-10127-1 (PMC5575162; doi:10.1038/s41598-017-10127-1)
Supplement: Supplementary file 1 — Statistical code used for generating the weighted gene co-expression network.doc [file 41598_2017_10127_MOESM1_ESM.pdf]

Analysis of temporal expression profiles after sciatic nerve injury by bioinformatic method

Yichong Zhang[a,1], *Yuanbo Zhan*[b,1], *Na Han*[a], *Yuhui Kou*[a], *Xiaofeng Yin*[a], *Peixun Zhang*[a,*]

[a] *Peking University People's Hospital, Beijing, China*

[b] *The Second Affiliated Hospital of Harbin Medical University, Harbin, China*

∗Co-corresponding authors at Peking University People's Hospital, 11 South Xizhimen Street, Beijing 100044, China.

Email -address : Prof.Peixun Zhang, zhangpeixun@bjmu.edu.cn

[1] These authors contributed equally to this work.

Statistical code for generating the weighted gene co-expression network

We provide the statistical code used for generating the weighted gene co-expression network analysis (WGCNA)[1]. All of the codes were modified by the protocol of WGCNA[1].

Microarray Data

The gene chip data of GSE33175[2] was obtained from National Center of Biotechnology Information Gene Expression Omnibus (GEO) database[3]. The more detail information about GSE33175 can refer to Wang et.al.[2]. Briefly, the platform used in GSE33175 was GPL7294 Agilent-014879 Whole Rat Genome Microarray 4x44K G4131F. The experiments were conducted on adult male Sprague-Dawley rats weighing 180–220 g. Proximal sciatic nerve tissues (0.5cm) were generated at 0h, 0.5h, 1h, 3h, 6h and 9h after sciatic nerve resection. Total RNA extracted from those tissues was used for cDNA array hybridization[2].The raw expression data were converted to log2 values and then normalized by using the quantiles normalization. In terms of spot filters, spots with intensity < 10 were removed. The replicate spots within an array were averaged. Moreover, the genes under any of the following conditions were excluded: percentile of the log-ratio variation in less than 75, percent of data missing or filtered out exceeds 50 %[4].

Data Reduction:

In order to minimize noise in the gene expression data set, several data filtering steps were taken. Multiple probes/probe sets were reduced to one per gene symbol by using most variable probe/probe set measured by Inter-Quartile Range (IQR) across arrays. This final filtering step yielded a count of 2211 genes for the experimental network construction.

#Loading expression data

```
# Load the WGCNA package
library(WGCNA);
# The following setting is important, do not omit.
options(stringsAsFactors = FALSE);
#Read in the female liver data set
femData = read.csv("GSE33175.csv");
# Take a quick look at what is in the data set:
dim(femData);
names(femData);
datExpr0 = as.data.frame(t(femData[, -1]));
names(datExpr0) = femData$UniqueID;
rownames(datExpr0) = names(femData)[-1];

gsg = goodSamplesGenes(datExpr0, verbose = 3);
gsg$allOK
```

# Checking data for excessive missing values and identi_cation of outlier microarray Samples

```
if (!gsg$allOK)
{
# Optionally, print the gene and sample names that were removed:
if (sum(!gsg$goodGenes)>0)
printFlush(paste("Removing genes:", paste(names(datExpr0)[!gsg$goodGenes], collapse = ", ")));
if (sum(!gsg$goodSamples)>0)
printFlush(paste("Removing samples:", paste(rownames(datExpr0)[!gsg$goodSamples], collapse
= ", ")));
# Remove the offending genes and samples from the data:
datExpr0 = datExpr0[gsg$goodSamples, gsg$goodGenes]
}

sampleTree = hclust(dist(datExpr0), method = "average");
# Plot the sample tree: Open a graphic output window of size 12 by 9 inches
sizeGrWindow(12,9)
#pdf(file = "Plots/sampleClustering.pdf", width = 12, height = 9);
par(cex = 0.6);
par(mar = c(0,4,2,0))
plot(sampleTree, main = "Sample clustering to detect outliers", sub="", xlab="", cex.lab = 1.5,
cex.axis = 1.5, cex.main = 2)

# Plot a line to show the cut
abline(h = 4, col = "red");
# Determine cluster under the line
clust = cutreeStatic(sampleTree, cutHeight = 4, minSize = 10)
table(clust)
# clust 1 contains the samples we want to keep.
```

```
keepSamples = (clust==1)
datExpr = datExpr0[keepSamples, ]
nGenes = ncol(datExpr)
nSamples = nrow(datExpr)
```

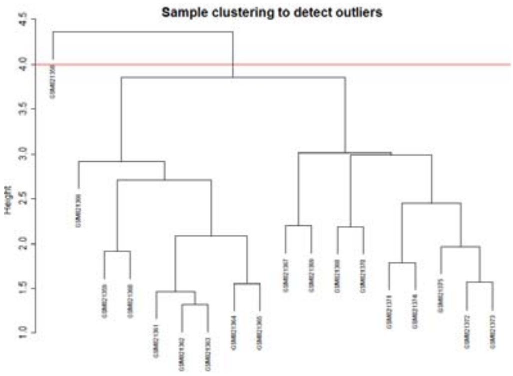


```
traitData = read.csv("ClinicalTraits.csv");
dim(traitData)
names(traitData)
# remove columns that hold information we do not need.
allTraits = traitData[, -1];
dim(allTraits)
names(allTraits)
# Form a data frame analogous to expression data that will hold the clinical traits.
femaleSamples = rownames(datExpr);
traitRows = match(femaleSamples, allTraits$ExpId);
datTraits = allTraits[traitRows, -1];
datTraits=data.frame(datTraits);
names(datTraits) = names(allTraits)[-1]
rownames(datTraits) = allTraits[traitRows, 1];
collectGarbage();
```

```
# Re-cluster samples
sampleTree2 = hclust(dist(datExpr), method = "average")
# Convert traits to a color representation: white means low, red means high, grey means missing entry
traitColors = numbers2colors(datTraits, signed = FALSE);
# Plot the sample dendrogram and the colors underneath.
plotDendroAndColors(sampleTree2, traitColors,
groupLabels = names(datTraits),
main = "Sample dendrogram and trait heatmap")
```

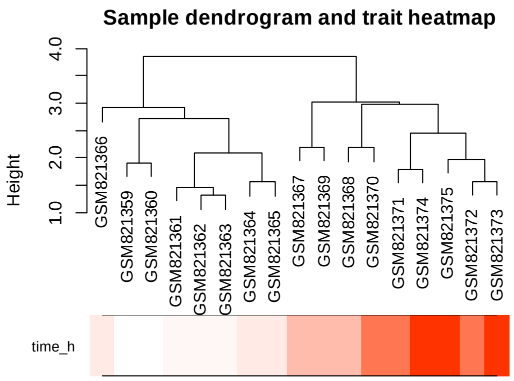


```
save(datExpr, datTraits, file = "FemaleLiver-01-dataInput.RData")
```

#Automatic network construction and module detection

```
# Choose a set of soft-thresholding powers
powers = c(c(1:10), seq(from = 12, to=20, by=2))
# Call the network topology analysis function
sft = pickSoftThreshold(datExpr, powerVector = powers, verbose = 5)
# Plot the results:
sizeGrWindow(9, 5)
par(mfrow = c(1,2));
cex1 = 0.9;
# Scale-free topology fit index as a function of the soft-thresholding power
plot(sft$fitIndices[,1], -sign(sft$fitIndices[,3])*sft$fitIndices[,2],
xlab="Soft Threshold (power)",ylab="Scale Free Topology Model Fit,signed R^2",type="n",
main = paste("Scale independence"));
text(sft$fitIndices[,1], -sign(sft$fitIndices[,3])*sft$fitIndices[,2],
labels=powers,cex=cex1,col="red");
# this line corresponds to using an R^2 cut-off of h
abline(h=0.80,col="green")
# Mean connectivity as a function of the soft-thresholding power
plot(sft$fitIndices[,1], sft$fitIndices[,5],
xlab="Soft Threshold (power)",ylab="Mean Connectivity", type="n",
main = paste("Mean connectivity"))
text(sft$fitIndices[,1], sft$fitIndices[,5], labels=powers, cex=cex1,col="red")
```

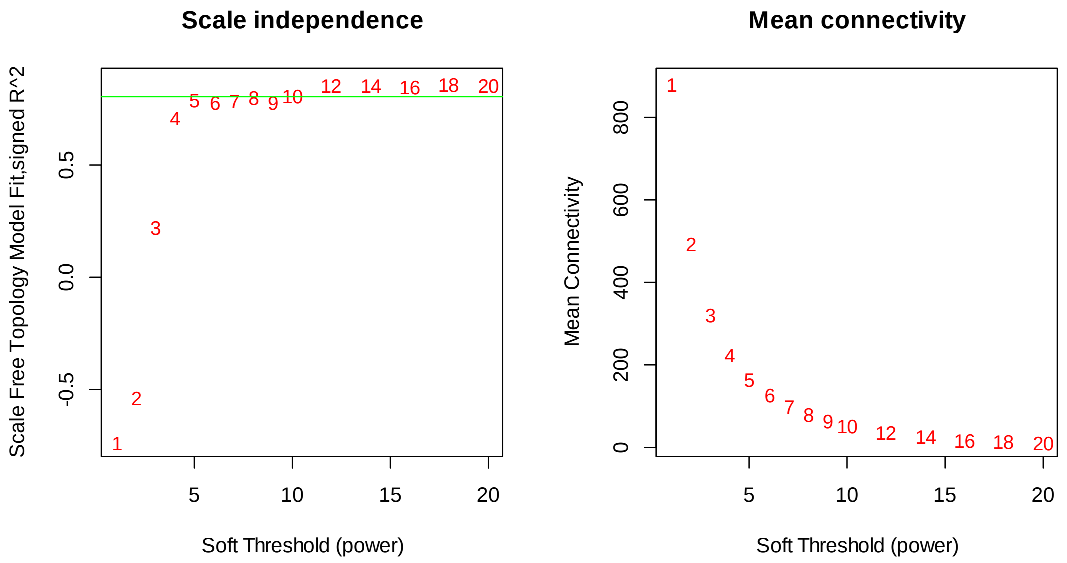


```
net = blockwiseModules(datExpr, power = 5,
TOMType = "unsigned", minModuleSize = 30,
reassignThreshold = 0, mergeCutHeight = 0.25,
numericLabels = TRUE, pamRespectsDendro = FALSE,
saveTOMs = TRUE,
saveTOMFileBase = "femaleMouseTOM",
verbose = 3)
table(net$colors)
  0   1   2   3   4   5   6   7   8   9
 65 661 534 301 185 173 115  82  55  40
# open a graphics window
sizeGrWindow(12, 9)
# Convert labels to colors for plotting
mergedColors = labels2colors(net$colors)
# Plot the dendrogram and the module colors underneath
plotDendroAndColors(net$dendrograms[[1]], mergedColors[net$blockGenes[[1]]],
"Module colors",
dendroLabels = FALSE, hang = 0.03,
addGuide = TRUE, guideHang = 0.05)

moduleLabels = net$colors
moduleColors = labels2colors(net$colors)
MEs = net$MEs;
geneTree = net$dendrograms[[1]];
save(MEs, moduleLabels, moduleColors, geneTree,
file = "networkConstruction-auto.RData")
```

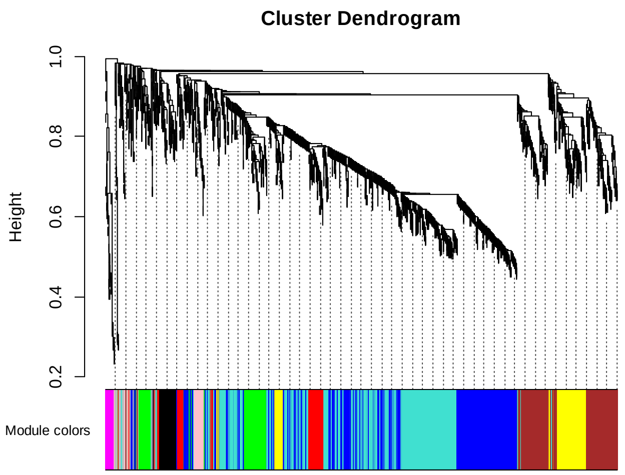


```
# Define numbers of genes and samples
nGenes = ncol(datExpr);
nSamples = nrow(datExpr);
# Recalculate MEs with color labels
MEs0 = moduleEigengenes(datExpr, moduleColors)$eigengenes
MEs = orderMEs(MEs0)
moduleTraitCor = cor(MEs, datTraits, use = "p");
moduleTraitPvalue = corPvalueStudent(moduleTraitCor, nSamples);

sizeGrWindow(10,6)
# Will display correlations and their p-values
textMatrix = paste(signif(moduleTraitCor, 2), "\n(",
signif(moduleTraitPvalue, 1), ")", sep = "");
dim(textMatrix) = dim(moduleTraitCor)
par(mar = c(6, 8.5, 3, 3));
# Display the correlation values within a heatmap plot
labeledHeatmap(Matrix = moduleTraitCor,
xLabels = names(datTraits),
yLabels = names(MEs),
ySymbols = names(MEs),
colorLabels = FALSE,
colors = greenWhiteRed(50),
textMatrix = textMatrix,
setStdMargins = FALSE,
cex.text = 0.5,
zlim = c(-1,1),
main = paste("Module-trait relationships"))
```

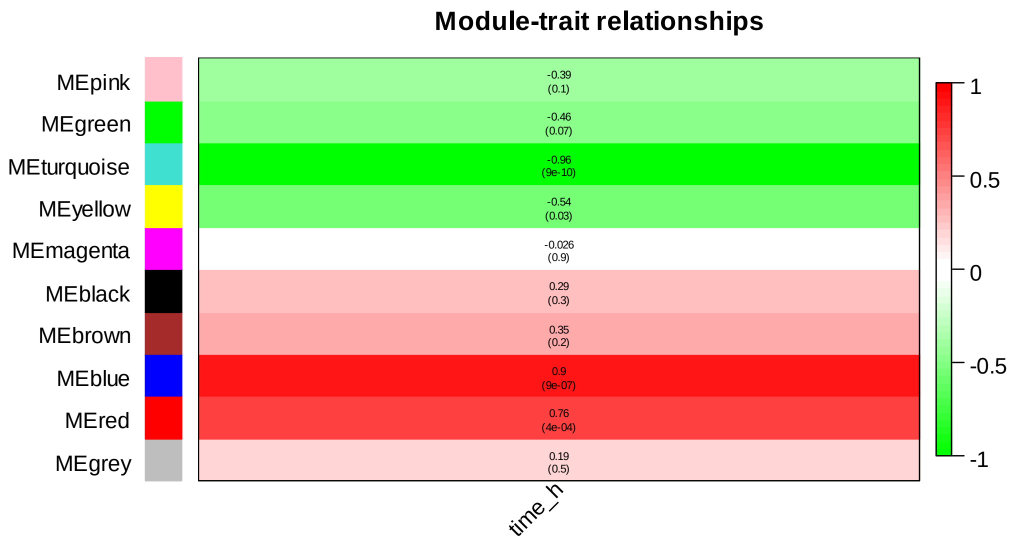


```
# Define variable time containing the time_h column of datTrait
time = as.data.frame(datTraits$time_h);
names(time) = "time"
# names (colors) of the modules
modNames = substring(names(MEs), 3)
geneModuleMembership = as.data.frame(cor(datExpr, MEs, use = "p"));
MMPvalue=as.data.frame(corPvalueStudent(as.matrix(geneModuleMembership), nSamples));
names(geneModuleMembership) = paste("MM", modNames, sep="");
names(MMPvalue) = paste("p.MM", modNames, sep="");
geneTraitSignificance = as.data.frame(cor(datExpr, time, use = "p"));
GSPvalue = as.data.frame(corPvalueStudent(as.matrix(geneTraitSignificance), nSamples));

module = "blue"
column = match(module, modNames);
moduleGenes = moduleColors==module;
sizeGrWindow(7, 7);
par(mfrow = c(1,1));
verboseScatterplot(abs(geneModuleMembership[moduleGenes, column]),
abs(geneTraitSignificance[moduleGenes, 1]),
xlab = paste("Module Membership in", module, "module"),
ylab = "Gene significance for time",
main = paste("Module membership vs. gene significance\n"),
cex.main = 1.2, cex.lab = 1.2, cex.axis = 1.2, col = module)
```

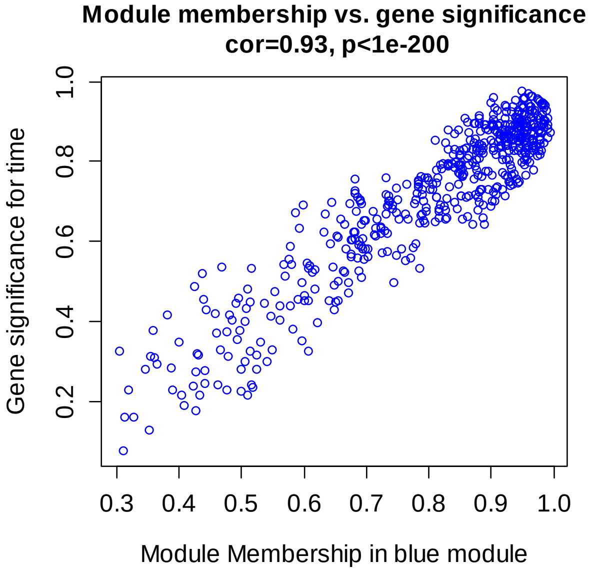


```
module = "turquoise"
column = match(module, modNames);
moduleGenes = moduleColors==module;
sizeGrWindow(7, 7);
par(mfrow = c(1,1));
verboseScatterplot(abs(geneModuleMembership[moduleGenes, column]),
abs(geneTraitSignificance[moduleGenes, 1]),
xlab = paste("Module Membership in", module, "module"),
ylab = "Gene significance for time",
main = paste("Module membership vs. gene significance\n"),
cex.main = 1.2, cex.lab = 1.2, cex.axis = 1.2, col = module)
```

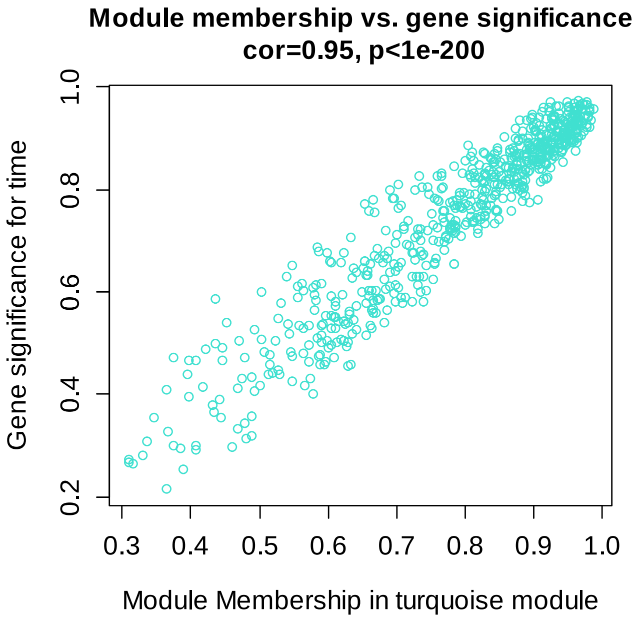

```
module = "yellow"
column = match(module, modNames);
moduleGenes = moduleColors==module;
sizeGrWindow(7, 7);
par(mfrow = c(1,1));
verboseScatterplot(abs(geneModuleMembership[moduleGenes, column]),
abs(geneTraitSignificance[moduleGenes, 1]),
xlab = paste("Module Membership in", module, "module"),
ylab = "Gene significance for time",
main = paste("Module membership vs. gene significance\n"),
cex.main = 1.2, cex.lab = 1.2, cex.axis = 1.2, col = module)
```

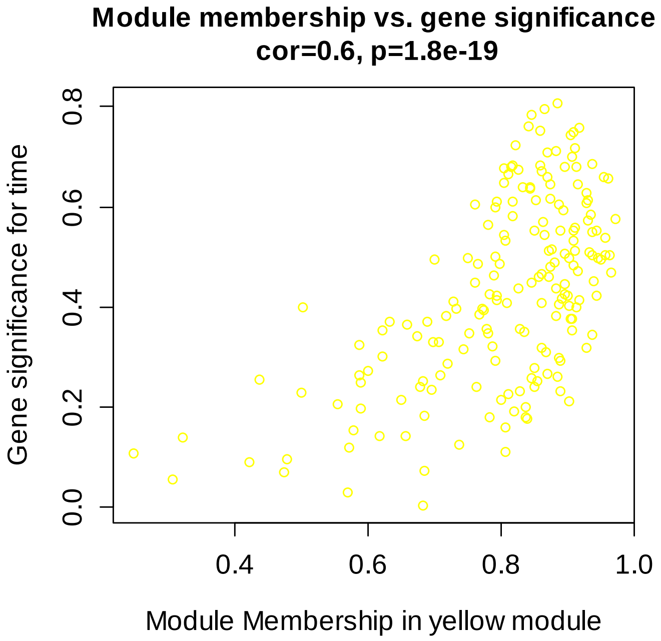


```
module = "red"
column = match(module, modNames);
moduleGenes = moduleColors==module;
sizeGrWindow(7, 7);
par(mfrow = c(1,1));
verboseScatterplot(abs(geneModuleMembership[moduleGenes, column]),
abs(geneTraitSignificance[moduleGenes, 1]),
xlab = paste("Module Membership in", module, "module"),
ylab = "Gene significance for time",
main = paste("Module membership vs. gene significance\n"),
cex.main = 1.2, cex.lab = 1.2, cex.axis = 1.2, col = module)
```

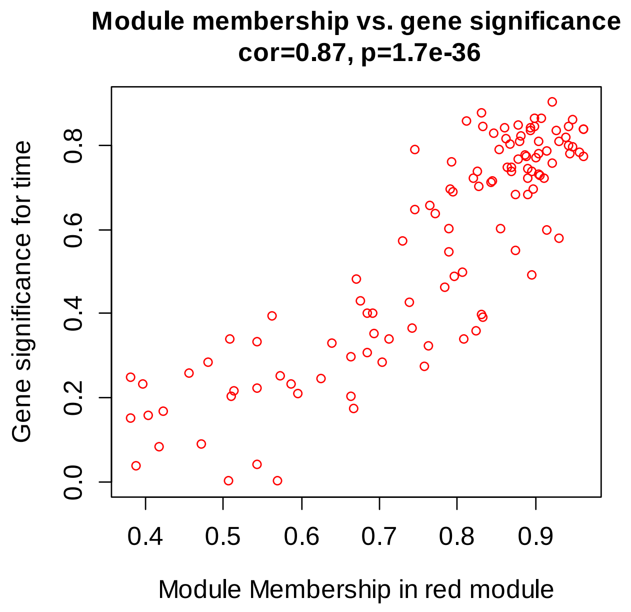


```
annot = read.csv(file = "GeneAnnotation.csv");
dim(annot)
names(annot)
probes = names(datExpr)
probes2annot = match(probes, annot$UniqueID)
# The following is the number or probes without annotation:
sum(is.na(probes2annot))
# Should return 0.
# Create the starting data frame
geneInfo0 = data.frame(UniqueID..Double.click. = probes,
geneSymbol = annot$Symbol[probes2annot],
EntrezID = annot$EntrezID[probes2annot],
moduleColor = moduleColors,
geneTraitSignificance,
GSPvalue)

# Order modules by their significance for time
modOrder = order(-abs(cor(MEs, time, use = "p")));
# Add module membership information in the chosen order
for (mod in 1:ncol(geneModuleMembership))
{
oldNames = names(geneInfo0)
geneInfo0 = data.frame(geneInfo0, geneModuleMembership[, modOrder[mod]],
MMPvalue[, modOrder[mod]]);
names(geneInfo0) = c(oldNames, paste("MM.", modNames[modOrder[mod]], sep=""),
paste("p.MM.", modNames[modOrder[mod]], sep=""))
}
```

```
write.csv(geneInfo0, file = " geneInfo0.csv", row.names = FALSE)
```

1 Langfelder, P. & Horvath, S. WGCNA: an R package for weighted correlation network analysis. *BMC bioinformatics* **9**, 559, doi:10.1186/1471-2105-9-559 (2008).

2 Wang, Y. *et al.* Gene network revealed involvements of Birc2, Birc3 and Tnfrsf1a in anti-apoptosis of injured peripheral nerves. *PloS one* **7**, e43436, doi:10.1371/journal.pone.0043436 (2012).

3 Davis, S. & Meltzer, P. S. GEOquery: a bridge between the Gene Expression Omnibus (GEO) and BioConductor. *Bioinformatics* **23**, 1846-1847, doi:10.1093/bioinformatics/btm254 (2007).

4 Simon, R. *et al.* Analysis of gene expression data using BRB-ArrayTools. *Cancer informatics* **3**, 11-17 (2007).
